# Gd/Mn Co-Doped CaBi_4_Ti_4_O_15_ Aurivillius-Phase Ceramics: Structures, Electrical Conduction and Dielectric Relaxation Behaviors

**DOI:** 10.3390/ma15175810

**Published:** 2022-08-23

**Authors:** Daowen Wu, Huajiang Zhou, Lingfeng Li, Yu Chen

**Affiliations:** 1 School of Mechanical Engineering, Chengdu University, Chengdu 610106, China; 2 Institute of Advanced Materials, Chengdu University, Chengdu 610106, China

**Keywords:** CaBi_4_Ti_4_O_15_, ion doping, electrical conduction, dielectric relaxation, oxygen vacancies

## Abstract

In this work, Gd/Mn co-doped CaBi_4_Ti_4_O_15_ Aurivillius-type ceramics with the formula of Ca_1-*x*_Gd*_x_*Bi_4_Ti_4_O_15_ + *x*Gd/0.2wt%MnCO_3_ (abbreviated as CBT-*x*Gd/0.2Mn) were prepared by the conventional solid-state reaction route. Firstly, the prepared ceramics were identified as the single CaBi_4_Ti_4_O_15_ phase with orthorhombic symmetry and the change in lattice parameters detected from the Rietveld XRD refinement demonstrated that Gd^3+^ was successfully substituted for Ca^2+^ at the A-site. SEM observations further revealed that all samples were composed of the randomly orientated plate-like grains, and the corresponding average grain size gradually decreased with Gd content (*x*) increasing. For all compositions studied, the frequency independence of conductivity observed above 400 °C showed a nature of ionic conduction behavior, which was predominated by the long-range migration of oxygen vacancies. Based on the correlated barrier hopping (CBH) model, the maximum barrier height *W_M_*, the dc conduction activation energy *E*_d__c_, as well as the hopping conduction activation energy *E*_p_ were calculated for the CBT-*x*Gd/0.2Mn ceramics. The composition with *x* = 0.06 was found to have the highest *E*_dc_ value of 1.87 eV, as well as the lowest conductivity (1.8 × 10^−5^ S/m at 600 °C) among these compositions. The electrical modules analysis for this composition further illustrated the degree of interaction between charge carrier *β* increases, with an increase in temperature from 500 °C to 600 °C, and then a turn to decrease when the temperature exceeded 600 °C. The value of *β* reached a maximum of 0.967 at 600 °C, indicating that the dielectric relaxation behavior at this temperature was closer to the ideal Debye type.

## 1. Introduction

It is well-known that bismuth layer structure ferroelectrics (BLSFs) are one of the important ferroelectric oxides, which have a general formula (Bi_2_O_2_) ^2+^ (A_m−1_B_m_O_3m+1_)^2−^, and their crystal structure composed of pseudo-perovskite blocks (A_m−1_B_m_O_3m+1_)^2−^ interleaved with bismuth oxide layers (Bi_2_O_2_)^2+^ along the *c*-axis [1,2,3]. Generally, A represents a tetravalent, pentavalent, and hexavalent ion (such as k^+^, Li^1+^, Zn^2+^, Ca^2+^, Sr^2+^, Cr^3+^, or La^3+^) [4], or the mixture of them. About B, it represents a tetravalent, pentavalent, or hexavalent ion (such as Ti^4+^, Ta^5+^, Nd^5+^). m is the number of BO_6_ octahedra in the pseudo-perovskite block (m = 1, 2, 3, 4, or 5) [5]. The CaBi_4_Ti_4_O_15_(CBT) shows the structure of A21am space group at room temperature, composing four perovskite-like TiO_2_ octahedron units stacked in between (Bi_2_O_2_)^2+^ layers.

For Aurivillius oxides, CBT ceramics attracted much attention from years ago, with simple preparation, transferring speed, a high fatigue strength, and low leakage current density, which are widely used in large equipment [6]. With the advancement of the aerospace industries, the research of high temperature piezoelectric acceleration sensor is urgent and necessary. Due to the high cure temperature (*T*_c_ = 790 °C) [7] and excellent fatigue resistance [8,9], Bismuth layered piezoelectric ceramics are widely used in piezoelectric acceleration sensors. However, the low piezoelectric property limits the application of Pure CBT, because its own layer structures limit the material transportation when sintering progress and spontaneous polarization (along *a-b* plane) [10,11,12]. Moreover, a low spontaneous polarization (*P*_s_) and higher coercive field (*E*_c_) requires higher polarization voltage, and high electrical conductivity leads to high leakage current [13]. Therefore, it is of certain significance to study the high-temperature conductivity of CBT for operating in high-temperature environment. For Aurivillius piezoceramics, it is necessary to study electrical resistivity and conduction behavior at high temperature. Until now, many studies about CBT have been reported that concentrated on the structures and how to improve the *T*_c_ or piezoelectricity [14,15,16]. For example, Gd^3+^ was found to reduce the leakage current and low loss [17]. Generally, the *p*-type conduction is mainly a conducting type for Aurivillius piezoceramics. As such, the dc conductivity can be reduced by donor doping [18]. There are few studies about the conduction behavior of CBT. For example, Xie et al. doped W^+^ into CaBi_4_Ti_4_O_15_ piezoceramics, the relaxation activation energy of the doped system was 1.45 eV, and its hopping conduction energy was 1.50 eV, while dc conduction energy was 1.39 eV [19], but the *d*_33_ of this system was only 17.8 pC/N. Many studies revealed that V^5^^+^, Nb^5^^+^, and W^6^^+^ can decrease the high-temperature conductivity and increase the piezoelectric properties of BLSF ceramics, since these donor-type substituted ions could release the distortion of the oxygen octahedral, as well as reduce the concentration of oxygen vacancies in the lattice [20,21,22]. This means that CBT ceramics may have two different conductive types at different temperatures. However, there are many studies on the conduction mechanism of bismuth layered oxide ceramics and various mechanisms are still not widely adopted. Therefore, it would be necessary to study the conductance mechanism of the CaBi_4_Ti_4_O_15_ ceramics, which is conducive to understanding of the microscopic motion energy of charge carriers [23].

In this work, a kind of Gd/Mn co-doped CaBi_4_Ti_4_O_15_ ceramics were prepared using the solid-state reaction method and the structures of samples were characterized by using XRD and SEM. The effects of Gd/Mn co-doping on the electrical conduction and dielectric relaxation behaviors of CaBi_4_Ti_4_O_15_ were studied in terms of the temperature dependent conductivity spectrum and electrical modulus analysis, with emphasis on the thermally activated motion of ionic defects, which predominates the dielectric behaviors at high temperature.

## 2. Experimental Section

### 2.1. Sample Preparation

A kind of Gd/Mn co-doped CaBi_4_Ti_4_O_15_ piezoceramics, the formula of Ca_1-*x*_Gd*_x_*Bi_4_Ti_4_O_15_+0.2wt%MnCO_3_ (abbreviated as CBT-*x*Gd/0.2Mn), were produced by the conventional solid-state reaction route. First of all, the CaCO_3_ of 99% purity, Gd_2_O_3_ of 99.99% purity, TiO_2_ of 99% purity, Bi_2_O_3_ of 99.99% purity, and MnCO_3_ of 99% purity (as raw materials) were weighed according to the stoichiometric ratio (CaCO_3_, Gd_2_O_3_, Bi_2_O_3_, TiO_2_, and MnCO_3_ produced in Chron Chemicals, Chengdu, China). These chemical compounds were balled for 6h with alcohol in planetary ball mill and calcined at 850 °C for 4 h in the muffle furnace. Then, the calcined powders, with 0.2 wt% MnCO_3_, were balled for another 12 h, pressed into discs, and sintered at 1050 ~ 1150 °C for 2h to obtain the ceramic chips. Lastly, Ag paste was painted on both sides of the CBT-*x*Gd/0.2Mn ceramics and fired at 700 °C for 10 min in air.

### 2.2. Sample Characterization

The phase structure for the samples were characterized through X-ray diffraction measurement (XRD, DX–2700B, Haoyuan Instrument, Dandong, China). The nature surfaces of the samples were observed using scanning electron microscope (SEM, Quanta FEG 250, FEI, Waltham, MA, USA). In the frequency range of 100~10^6^ Hz, the dielectric properties were measured (room temperature ~700 °C) by an LCR meter (TH2829A, Tonghui Electronic, Changzhou, China) and the high temperature conductivity and complex impedance behavior were analyzed.

## 3. Results and Discussion

### 3.1. Phase Structures

**Figure 1:**
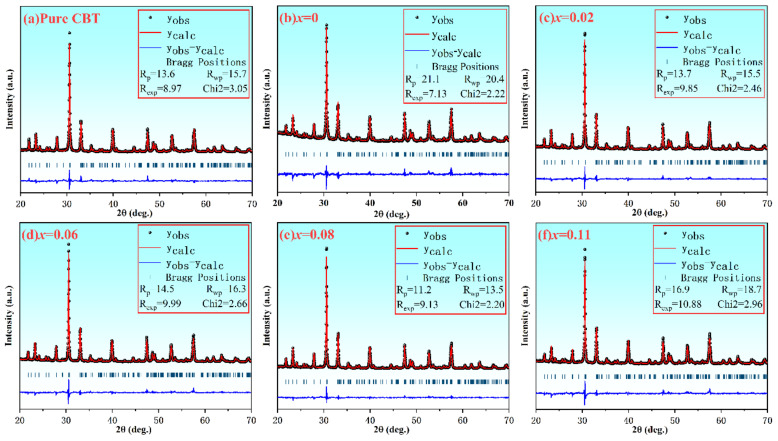
Rietveld analysis of XRD patterns of the CBT−*x*Gd/0.2Mn ceramics measured at room temperature.

The XRD patterns of the pure CBT and CBT-*x*Gd/0.2Mn ceramics were performed as shown in Figure 1. It indicated the X-ray diffraction peak of the Pure CBT and the CBT-*x*Gd/0.2Mn ceramics were consistent with the JCPDS card No.52-1640. Furthermore, all samples were orthorhombic in structure and A21am in space group. There is no other phase from the XRD pattern results of the CBT-*x*Gd/0.2Mn ceramics, which indicated co-doping Gd/Mn formed a complete solid solution with CaBi_4_Ti_4_O_15_; the strongest diffraction peak of the CBT-*x*Gd/0.2Mn ceramics was (1 1 9) peak, which was consistent with the strongest diffraction peak (1 1 2m+1) of BLSF ceramics [24,25]. Compared with the pure CBT, CBT-*x*Gd/0.2Mn ceramics showed a smaller cell volume (*V*) from the Table 1 of the cell paraments, and the results revealed Gd/Mn could reduce the grain size, which was valuable to increase the piezoelectricity. The amount of Gd/Mn co-doping increased had little change to the orthorhombic distortion (*a*/*b*).

### 3.2. Microstructures

**Figure 2:**
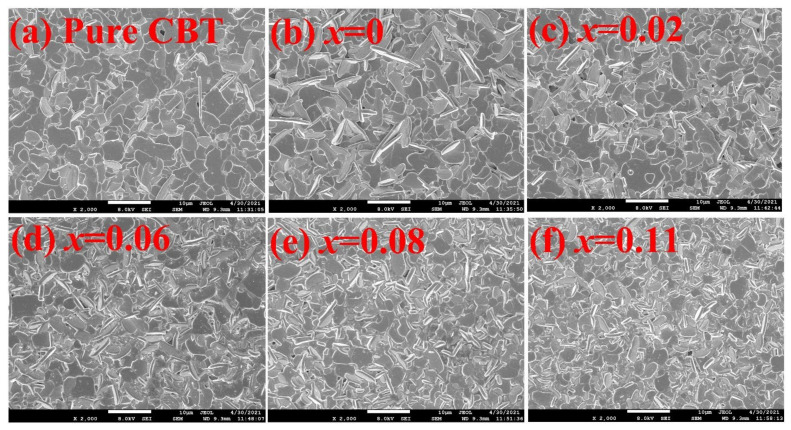
SEM images focused on the original surfaces of the CBT−*x*Gd/0.2Mn ceramics.

Figure 2 shows the SEM images of the CBT-*x*Gd/0.2Mn ceramics of the original surface. It can be seen from Figure 2 that the CBT-*x*Gd/0.2Mn ceramics presented a dense structure composed of many plate-like grains with random orientation. Such a special morphology was formed due to the structurally highly anisotropic grain growth, which had a much higher grain growth rate in the direction perpendicular to the *c*-axis of the BLSFs crystal [26]. Horn et al [27] reported that the (0 0 *l*)-type planes of the BLSFs crystal possessed a lower surface energy, which developed predominantly during sintering. Although the plate-like grains with their *c*-axis were normally oriented to the major surface preferred to grow up in the BLSFs ceramics, the grain orientation was random in the CBT-*x*Gd/0.2Mn piezoceramics, as the ceramics were fabricated by pressure-less sintering.

**Figure 3:**
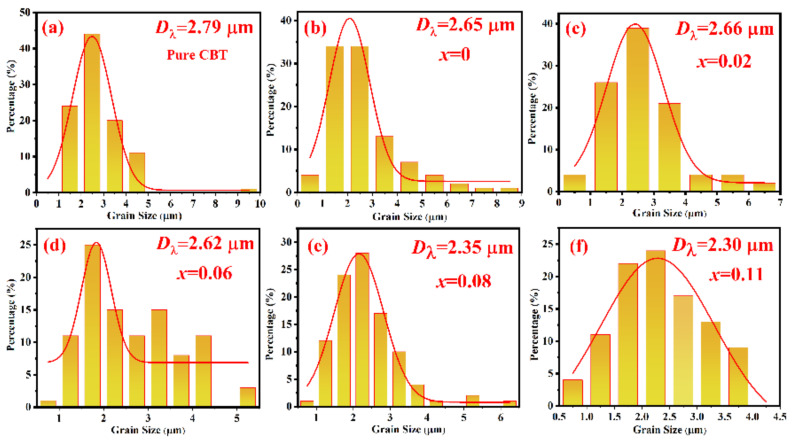
Grain size distribution of the CBT−*x*Gd/0.2Mn ceramics derived from SEM images.

In order to explore the grain characteristics of the CBT-*x*Gd/0.2Mn piezoceramics quantitatively, the linear intercept method (performed by the Nano Measurer software) was used to obtain the grain size distribution from SEM images, the results were shown in Figure 3. When *x* increased from 0 to 0.11, the average grain size (*D*_λ_) gradually decreased from 2.65 μm to 2.30 μm, and the corresponding size distribution became more inhomogeneous. Among the CBT-*x*Gd/0.2Mn piezoceramics, the composition with *x* = 0.11 had the smallest grain size (*D*_λ_ = 2.30 μm) and the widest size distribution; such refined grains and compact structure could reduce the oxygen vacancy concentration and improve the activation energy of grain boundary, so as to provide a higher poling electric field to the ceramic. Alternatively, an obvious grain refinement, which was accompanied by more random grain orientation, occurred in those samples with *x* ≧ 0.08, indicating that enough Gd^3+^ entering into the A-site of perovskite unit would influence the growth behavior of ceramic grains. This phenomenon could be attributed to the reduced boundary energy for grain boundary migration or the increased activating energy for ion migration [28]. 

### 3.3. Electrical Conduction Behaviors

**Figure 4:**
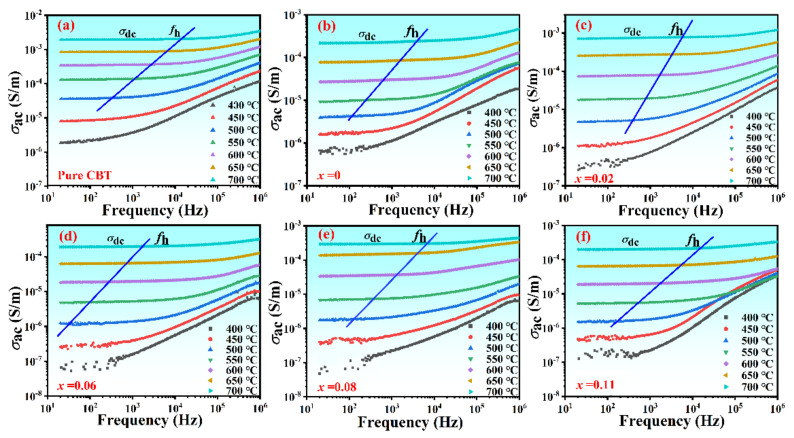
Frequency dependence of AC conductivity of the CBT−*x*Gd/0.2Mn ceramics measured at different temperatures.

The growth of the ferroelectric phase and the movement of charge carriers are affected by conductivity to a certain extent. The study of conductivity not only helps to clarify the influence of conductivity on domain structure and its motion, but also helps to clarify the properties of carriers. The AC conductivity (*σ*_ac_) of CBT-*x*Gd/0.2Mn ceramics was studied to better understand the relaxation-conduction behaviors of the system. The AC conductivity *σ*_ac_ of dielectrics could be calculated using the following relation

(1) $$\sigma_{ac}=\omega \varepsilon_{0}\varepsilon_{r}•\tan\delta$$

where *ω* is the frequency of the applied electric field, *ε*_0_ is the permittivity of free space, *ε*_r_ is the dielectric constant, and tan *δ* is the dissipation factor. Frequency dependence of *σ*_ac_ at various temperatures is shown in Figure 4.

It can be seen from Figure 4a–f that the conductivity spectra of all samples exhibited the following characteristics: (i) The conductivity curved at lower temperatures and higher frequencies were frequency dependent, whereas at higher temperatures and lower frequencies these plots showed the frequency independence. (ii) The characteristic frequency (*f*_h_ as marked by the arrow), where the conductivity became dependent on frequency and independent on frequency, moved to a higher frequency with the temperature increasing. (iii) In the high-frequency region, the dispersion of conductivity was less and all the curves tended to merge with a single slope. The peak observed at the low frequency was due to the application of low frequency AC electric field on the high concentration doped samples, which caused the de-coupling of a large number of internal defect dipoles, resulting in a relaxed dielectric loss peak. For the perovskite-type ferroelectrics, the increase of conductivity with increasing of frequency and temperature was usually attributed to the hopping of charge carriers through the barrier or the moving of ionic defects as space charges [29,30].

The frequency dependence of conductivity has long been found to obey the following Jonscher’s power law [31]:

(2) $$\sigma_{ac}=\sigma_{0}+A\left(T\right)\omega^{s\left(T\right)}$$

where *σ_ac_* is the AC conductivity, *σ*_0_ is the frequency independent (i. e. DC) conductivity, which can be obtained by extrapolating these plots in the low-frequency region, *ω* (=2*πf*) is the angular frequency of the AC electric field in the high-frequency region, *A* is a characteristic parameter assigning the polarization strength, and *s* is a dimensionless exponent to evaluate the degree of interaction between mobile charge carriers and surrounding lattice. Both *A* and the exponent *s* are the temperature and material intrinsic property dependent constants, which can be obtained from the fitting of the frequency dependence of conductivity according to Equation (2). The frequency and temperature dependance of ac conductivity of CBT-*x*Gd/Mn ceramics had been carried out by Jonscher’s theory, as shown in Figure 4a–f.

**Figure 5:**
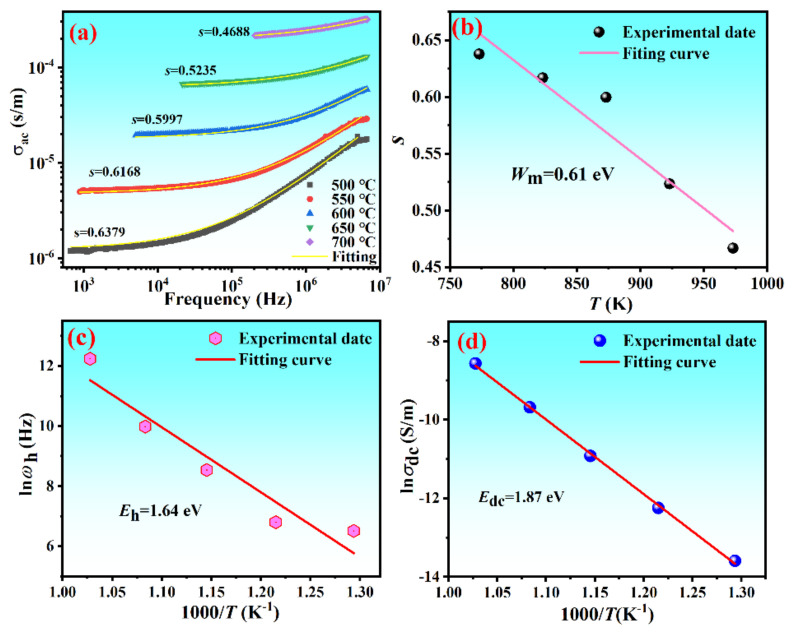
Fitting for the temperature and frequency dependence of conduction parameters of the composition with *x* = 0.06.

Ion concentrations and ion jump frequency have an influence on the conductivity of ion conductivity. The ac conductivity can be obtained by Equation (3), and the *ω*_p_ and dc conductivity are calculated by Arrhenius Equations (4) and (5), respectively [32]:

(3) $$\omega_{p}={\left(\frac{\sigma_{ac}}{A}\right)}^{1/s}$$

(4) $$\omega_{p}=\omega_{0}\exp\left(-E_{h}/k_{B}T\right)$$

(5) $$\sigma_{dc}=\sigma_{0}\exp\left(-E_{dc}/k_{B}T\right)$$

where *ω_p_* is the hopping angular frequency, *k*_B_ is the Boltzmann constant, and *T* is the absolute temperature (K). Both *ω*_0_ and *σ*_0_ are the pre-exponential factor. *E*_h_ and *E*_dc_ are the activation energy of hopping conduction and dc conduction activation energy, respectively. Figure 5a shows the fitting of the *σ_ac_*-*f* curves for the composition with *x* = 0.06 measured at different temperatures (500~700 °C). It can be seen that the values of s decreased with temperature increasing, indicating that the electrical conduction was a thermally activated process, which agreed with the correlated barrier hopping (CBH) model [33]. The result that s < 1 (s = back hop rare/site relaxation rate, which was defined by the jump relaxation model [34]) indicated that the time of the charge carriers returning to initial position was longer than its relaxation times. Oxygen vacancy and bismuth vacancy may cause the decrease of s value at high temperature, and the free movement of these charge carriers reduces the probability of back hoping rate.

According to the CBH model, the hopping of electrons between the charged defects was limited in finite clusters, where they were bound to various defects different from the free carriers. The conduction could be attributed to the short-range hopping of localized charge carriers over trap sites separated by energy barriers of different heights. The maximum barrier height *W*_M_, defined as the energy required to remove the electrons completely from one site to another [35], could be evaluated by using the following equation:

(6) $$s=1-\frac{6k_{B}T}{W_{M}}$$

Figure 5b shows the fitting of the *s*-*T* curve for the composition of *x* = 0.06, according to the equation above. Before 600 °C, the obtained value of *W*_M_ (~0.61 eV) agreed well with the activation energy (*E*_a_ = 0.3~0.5 eV [36]) of single-ionized oxygen vacancies (V_O_^•^), which confirmed the single-polaron hopping of electrons from the localized oxygen vacancies to the double-ionized oxygen vacancies (V_O_^•^→V_O_^••^+*e*′) in this material. At low frequencies, electrons underwent successive and successful hopping motions for long time periods, but the ratio between successful and unsuccessful hopping, along with the relaxation of the surrounding charged carriers caused the dispersion of conductivity at high frequencies [37]. In the high-frequency region, the conductivity increase d with the increase of frequency, which may have been due to the hopping of charge carriers in finite clusters. The frequency at which the change in a slope occurs is known as the hopping frequency *ω*_h_ (=2*πf*_h_), which obeyed the Arrhenius relation. The plots of *lnf*_p_ vs. 1000/*T* was depicted for the composition of *x* = 0.06 in Figure 5c and the value of *E*_h_ was calculated from the slope of the fitting line according to Equation (4). The *E*_h_ value is calculated to be 1.64 eV for the sample.

At low frequencies and high temperatures, the long-range migration of charge carriers contributed to the DC conductivity (*σ*_dc_). With the increase of temperature, an increase in charge carrier due to thermal ionization resulted in an increased *σ*_dc_. Therefore, the temperature dependence of DC conductivity could be described by the Arrhenius relation as Equation (5). Figure 5d shows the plots of *lnσ*_dc_ vs. 1000/*T* and the value of *E*_dc_ was estimated from the fitting of the *σ*_dc_~*T* curve based on the equation above. The fitting result estimated the value of *E*_dc_ to be 1.87 eV for the composition. Here, a small difference between the values of *E*_dc_ and *E*_h_ in the same temperature region indicated the similar type of localized charge carriers responsible for the DC and AC conduction. However, because the activation energy for the conduction process was the sum of diffusion activation (*E*_dc_) and the formation energy (*E*_h_) of charge carriers. *E*_h_ < *E*_dc_, indicated that the hopping distance of charge carriers (usually limited in a unit-cell) was always shorter than their diffusing distance (including bulk/intragranular diffusion and grain boundary diffusion).

Table 2 listed the electrical conduction parameters of the CBT-*x*Gd/0.2Mn ceramics calculated according to the method above. As for the pure CBT ceramic, the estimated value of DC activation energy (*E*_dc_ = 1.28 eV) was less than half the band gap value of CBT (*E*_g_ = 3.36 eV), indicating an extrinsic conduction process existing in the ceramic [38]. The activation energy was generally associated with the acceptor or donor levels. For the CBT-*x*Gd/Mn ceramics, the values of *W*_M_, *E*_p_ and *E*_dc_ presented a mostly consistent varying trend with the doping content of Gd. The estimated value of *E*_dc_ was found to increase from 1.31 eV to 1.87 eV with an increase in *x* from 0 to 0.06 and then showed a decrease to 1.59 eV till *x* = 0.11. The composition of *x* = 0.06, *E*_dc_ reached to the maximum value of 1.87 eV (associated with a relatively high *E*_h_ value of 1.64 eV), so that the composition with *x* = 0.06 could obtain the lowest *σ*_dc_ value of 1.8 × 10^−^^5^ S/m among the CBT-*x*Gd/Mn ceramics.

In References. [7,15], Z-Y. Shen et al. prepared a kind of Nd/Mn co-doped CBT ceramics, where the activation energy (*E*_a_ = 1.2–1.3 eV) in the temperature range 300~600 °C were suggested to be closed to the high temperature dc conductivity activation energy (*E*_dc_) reported for other BLSF ceramics, which was predominated by the conduction mechanism of intrinsic charge carriers. As compared with his works, the Gd/Mn co-doped CBT ceramics prepared in this work presented a higher activation energy (*E*_dc_ = 1.31–1.87 eV). At high temperatures, when the intrinsic conduction predominates the material, the nominal activation was the sum of diffusion activation (*E*_d_) and the formation energy of charge carrier (*E*_f_). Therefore, a higher activation energy observed in our material may be owing to the different doping effect between Gd and Nd in the CBT lattice. Considering the substitution of Gd^3+^ and Nd^3+^ for Ca^2+^ at A-site, a stronger Gd–O bonds compared to Nd–O bonds might induce an increase in the formation energy of oxygen vacancies.

It was well known that the primitive BLSFs were usually not stoichiometric, since that contained amounts of inherent defects, such as oxygen vacancies and bismuth vacancies, et al. This was owing to that the unavoidable volatilization of Bi_2_O_3_ during the high-temperature sintering of ceramics would produce the complexes of bismuth and oxygen vacancy in the (Bi_2_O_2_)^2+^ layers. Therefore, the doubly positively charged defects, oxygen vacancy V_O_^••^, was considered to be the most mobile intrinsic ionic defect in the perovskite-type ferroelectrics. Their long-range migration in the octahedra of any perovskite structure, which was evidenced through greatly enhanced conductivity and activation energy of ~1 eV [39], contributed to the intrinsic ionic conduction in the temperature region of ~300 °C to ~700 °C [39].

Alternatively, according to the experimental data presented in Table 2, the variation of conductivity of the CBT-*x*Gd/0.2Mn ceramics with the doping content of Gd (*x*) did not seem to be regular. The lowest conductivity value at a high temperature (600 °C) was observed at *x* = 0.06. For a single phase material with a homogenous microstructure, the electrical conductivity, σ, depended on both the concentration (*n*) and mobility (*μ*) of charge carriers and obeyed the following simplified equation *σ* = *nqμ*. Here, *q* was the number of charges per charge carrier. With increasing *x*, although the oxygen vacancy concentration tended to be decreased by the donor substitution of Gd^3+^ for Ca^2+^, the change in the mobility of oxygen vacancies could not be determined. As a result, the conductivity value at a high temperature was not expected to present a regular trend with change in *x* for the CBT-*x*Gd/0.2Mn ceramics. Similar to the investigation on the doping effect in layer structured SrBi_2_Nb_2_O_9_ ferroelectrics [40], the experimental results in this work suggested that the doping effects of A-site (Ca/Gd) on the dc conduction were complex, and further analysis is required to achieve a better understanding.

### 3.4. Electrical Impedance Analysis

**Figure 6:**
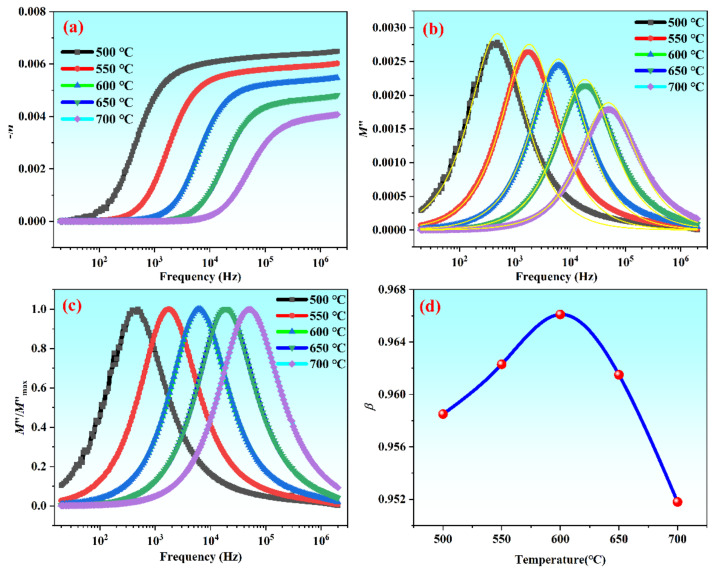
Electrical modulus spectroscopy of the composition with *x* = 0.06 measured at different temperatures: (**a**) −*M*′; (**b**) *M*″; (**c**) *M*″*/M*″_max_; (**d**) temperature dependence of *β*.

To further study the dielectric relaxation behavior of the CBT-*x*Gd/0.2Mn ceramics, Figure 6 shows the electrical modulus of *x* = 0.06 at a different temperature. The complex electrical modulus (M*) was calculated from the electrical modulus measured based on the following equations:

(7) $$M^{*}=M\prime +jM\prime \prime =j\omega C_{0}Z^{*}$$

(8) $$M\prime =j\omega C_{0}Z^{\prime \prime }$$

(9) $$M\prime \prime =j\omega C_{0}Z^{\prime }$$

(10) $$Z^{*}=Z^{\prime }-jZ^{\prime }$$

where *C*_0_ is the capacitance of free apace given by *C*_0_ = *ε_0_A/d* [41], Z^*^ is the complex electrical impedance, and *Z*′ and *Z*″ is its real part and imaginary part, respectively. It is shown that the *M*′ values increased quickly with frequency rising at low temperature, and slowly increased gradually with frequency increasing. Moreover, the reason for the relative dispersion in low frequency region may be related to short range hopping of charge carriers and lack of recovery energy [42]. Besides, only one single peak can be seen from Figure 6b, which results from only the grain response was observed [19] at the temperature and frequencies. *M*″ increased sharply and reached the top may be related to both grain size and grain boundary relaxation. However, the peak value of *M*″ declined with the increase of temperature, which demonstrated the relaxation deviated from Debye-type relaxation. The results showed that the ions move in a hopping manner along with other related carriers [43].

To further clarify the dielectric relaxation mechanism, the electric modules *M*″ were normalized to research the relaxation process (Figure 6c). The shape of the curves were asymmetrical and higher than Debye-type relaxation. The Bergman formula [44] can explain the phenomenon:

(11) $$M^{\prime \prime }\left(\omega \right)=\frac{M{\prime \prime }_{max}}{1-\beta +\frac{\beta }{1+\beta }{\left[\beta \left(\omega_{max}/\omega \right)+\left(\omega /\omega_{max}\right)\right]}^{\beta }}$$

where *M*″_max_ is the maximum value of *M*″, *ω*_max_ is the angular frequency corresponding to *M*″_max_, and *β* indicates the ideal Debye model—the closer the *β* value is to 1, the more it consistent with Debye-type relaxation [41]. *β* tended to increase to 1 as the temperature rose from 500 °C to 600 °C (Figure 6d), which showed that the relaxation type of the sample was closer to the Debye-type relaxation. A higher value of *β* indicated a weaker interaction between charge carriers. However, *β* began to decrease with increasing temperature at 600 °C, showing the dielectric relaxation behavior began to deviate from the Debye-type relaxation, which may be owing to the increased leakage current at the temperature above 600 °C. This phenomenon was consistent with that the peak value of *M*″ was found to decrease faster when the temperature exceeded 600 °C.

## 4. Conclusions

Gd/Mn co-doped CaBi_4_Ti_4_O_15_ (CBT-*x*Gd/0.2Mn, *x* = 0, 0.02, 0.06, 0.08, 0.11) ceramics were synthesized by the solid-state reaction method. The structures, electrical conduction, and dielectric relaxation behaviors of CBT-*x*Gd/0.2Mn ceramics were studied. Some main results were obtained as follows:

(1) CBT-*x*Gd/0.2Mn possessed a typical orthorhombic structure and the induced Gd^3+^ succeeded in substituting for Ca^2+^ at A-site. A dense microstructure composed of plate-like grains were observed in the prepared ceramics and the introduction of Gd^3+^ led to the decrease in the average grain size.
(2) The ionic conduction behavior in the high temperature region was related to the long-range migration of oxygen vacancies. The composition with *x* = 0.06 was found to have the highest *E*_dc_ value of 1.87 eV, as well as the lowest conductivity (1.8 × 10^−5^ S/m at 600 °C) among these compositions.
(3) The values of *β* (the degree of interaction between charge carriers) first increased and then decreased with increasing the temperature from 500 °C to 700 °C, the maximum value of 0.967 occurring at 600 °C suggested the dielectric relaxation behavior to be very close to the ideal Debye type.

## Figures and Tables

**Table 1:** Lattice parameters of the CBT-*x*Gd/0.2Mn ceramics.

	aBi_4_Ti_4_O_15_	CBT-*x*Gd/0.2Mn				
		*x* = 0	*x* = 0.02	*x* = 0.06	*x* = 0.08	*x* = 0.11
*a* (Å)	5.431	5.428	5.426	5.428	5.426	5.426
*b* (Å)	5.412	5.408	5.409	5.410	5.408	5.409
*c* (Å)	40.73	40.71	40.71	40.74	40.73	40.74
*V* (Å^3^)	1197.2	1194.9	1195.0	1196.2	1195	1195.5
*a*/*b*	1.0036	1.0036	1.0032	1.0034	1.0032	1.003

**Table 2:** Electrical conduction parameters of the CBT-*x*Gd/0.2Mn ceramics.

		CBT-*x*Gd/0.2Mn				
	CaBi_4_Ti_4_O_15_	*x* = 0	*x* = 0.02	*x* = 0.06	*x* = 0.08	*x* = 0.11
*W*_M_ (eV)	0.38	0.59	1.24	0.96	0.61	0.47
*E*_h_ (eV)	1.22	0.93	1.21	1.64	1.69	1.29
*E*_dc_ (eV)	1.28	1.31	1.65	1.87	1.72	1.59
*σ*_dc_ (S/m,600 °C)	3.39 × 10^−4^	2.69 × 10^−5^	3.1 × 10^−5^	1.8 × 10^−5^	3.34 × 10^−5^	1.90 × 10^−5^

## Data Availability

Not applicable.
